# The Effects of Methylphenidate on Goal-directed Behavior in a Rat Model of ADHD

**DOI:** 10.3389/fnbeh.2015.00326

**Published:** 2015-11-25

**Authors:** Joman Y. Natsheh, Michael W. Shiflett

**Affiliations:** ^1^Center for Molecular and Behavioral Neuroscience, Rutgers, The State University of New Jersey, NewarkNJ, USA; ^2^Palestinian Neuroscience Initiative, Faculty of Medicine, Al-Quds UniversityJerusalem, State of Palestine; ^3^Department of Psychology, Rutgers, The State University of New Jersey, NewarkNJ, USA

**Keywords:** attention deficit hyperactivity disorder, spontaneously hypertensive rats, Wistar–Kyoto rats, goal-directed behavior, methylphenidate

## Abstract

Although attentional and motor alterations in Attention Deficit Hyperactivity Disorder (ADHD) have been well characterized, less is known about how this disorder impacts goal-directed behavior. To investigate whether there is a misbalance between goal-directed and habitual behaviors in an animal model of ADHD, we tested adult [P75–P105] Spontaneously Hypertensive Rats (SHR; ADHD rat model) and Wistar–Kyoto rats (WKY), the normotensive control strain, on an instrumental conditioning paradigm with two phases: a free-operant training phase in which rats separately acquired two distinct action–outcome contingencies, and a choice test conducted in extinction prior to which one of the food outcomes was devalued through specific satiety. To assess the effects of Methylphenidate (MPH), a commonly used ADHD medication, on goal-directed behavior, we injected rats with either MPH or saline prior to the choice test. Both rat strains acquired an instrumental response, with SHR responding at greater rates over the course of training. During the choice test WKY demonstrated goal-directed behavior, responding more frequently on the lever that delivered, during training, the still-valued outcome. In contrast, SHR showed no goal-directed behavior, responding equally on both levers. However, MPH administration prior to the choice test restored goal-directed behavior in SHR, and disrupted this behavior in WKY rats. This study provides the first experimental evidence for selective impairment in goal-directed behavior in rat models of ADHD, and how MPH acts differently on SHR and WKY animals to restore or impair this behavior, respectively.

## Introduction

Attention Deficit Hyperactivity Disorder (ADHD) is one of the most prevalent psychiatric disorders, which is typically diagnosed in childhood and can continue to adolescence and adulthood. It is characterized by developmentally inappropriate symptoms of inattention, impulsivity, and hyperactivity (Castellanos and Tannock, 2002; Barkley, 2005). The neurobiological underpinnings of ADHD are not well established; however, dopaminergic hypofunction is thought to play an important role in the etiology of this disorder (Hynd et al., 1993; Gill et al., 1997; Waldman et al., 1998; Russell, 2003; Bush et al., 2005; Sagvolden et al., 2005b). Consistent with this notion, ADHD symptoms are reduced in response to drugs that increase dopamine signaling, such as Methylphenidate (MPH) (Ritalin©), a psychostimulant that preferentially blocks the reuptake of catecholamines, including dopamine (DA) and norepinephrine (NE), in both the striatum and prefrontal cortex (Mehta et al., 2000, 2004; Schiffer et al., 2006; Heal et al., 2009).

The spontaneously hypertensive rat strain (SHR), a rat model bred from progenitor Wistar–Kyoto rats (WKY; Okamoto and Aoki, 1963), is the most widely accepted rodent model of ADHD (Sagvolden et al., 1993; Sagvolden, 2000; Davids et al., 2003). SHR rats show deficits in sustained attention, motor impulsiveness, and hyperactivity in a novel environment (Knardahl and Sagvolden, 1979; Sagvolden et al., 1992, 2005b; Wultz and Sagvolden, 1992; Sagvolden, 2000). Further, SHR rats display reduced DA signaling and increased DA transporter (DAT) expression, similar to ADHD patients (Russell et al., 1995; Russell, 2003; Heal et al., 2008; Roessner et al., 2010). Likewise, MPH corrects attentional and motor impairments in SHR rats, lending further support for SHR rats as a model of human ADHD (Sagvolden et al., 2005a,b; Kantak et al., 2008).

Although attentional and motor alterations in ADHD have been well characterized, less is known about how this disorder impacts goal-directed behavior. Maze performance of SHR rats suggests they preferentially use response strategies to guide behavior in spatial tasks (Clements and Wainwright, 2006; Clements et al., 2007; Kantak et al., 2008). Here we employ operant conditioning procedures that allow us to more precisely distinguish goal-directed from stimulus–response (habitual) action control. Contemporary theories of action control suggest that two processes guide action selection: (1) a goal-directed system based on current knowledge of action–outcome contingencies and (2) a habit system based on acquired stimulus–response associations (Dickinson, 1985). Operant paradigms, such as pressing a lever to receive a food reward, provide means of assessing goal-directed and habitual action control. For example, changes in operant behavior in response to changes in either outcome value or the action–outcome contingency reflect goal-directed action control, whereas lack of sensitivity to these changes likely reflects habitual action control. Behavior of SHR rats has yet to be assessed using these paradigms.

Studying goal-directed behavior in ADHD will advance our understanding of the brain networks involved in reward processing and contingency learning in ADHD, thereby revealing new mechanisms and potential treatments for this disorder (Griffiths et al., 2014). In the context of reinforcement mechanisms, Tripp and Wickens (2008, 2009) propose that one factor underlying ADHD is diminished anticipatory DA cell firing. They suggest that, in ADHD, the transfer of DA signals to cued rewards fails to develop normally, especially late in learning, which leads to more rapid extinction of the behavioral response and loss of behavioral control (Tripp and Wickens, 2008, 2009; Furukawa et al., 2014). These studies, while not directly examining goal-directed and habitual action control, suggest that these processes may be altered in ADHD. On the other hand, some studies suggest that DA levels in the prefrontal cortex and the dorsomedial striatum are critical for action control as measured by contingency degradation, but not outcome devaluation test (Naneix et al., 2009; Lex and Hauber, 2010).

In the present study we examined goal-directed action control in SHR rats using an instrumental learning paradigm. Although previous research has shown overactive instrumental responding in SHR rats that was corrected by MPH, it is not known whether animals performed responses in a goal-directed or habitual manner (Sagvolden et al., 1993). We used outcome devaluation and contingency degradation paradigms to probe goal-directed behavior in adult SHR and WKY rats. We also examined the effects of an acute dose of MPH on choice behavior following outcome devaluation in SHR and WKY rats. Our findings illustrate that SHR rats are predominated by habitual action control; however, MPH can restore goal-directed control in these rats. In contrast, MPH impaired goal-directed behavior in control rats that previously showed intact behavior following saline injection.

## Materials and Methods

### Subjects and Apparatus

Twenty nine male adult (P75–P105) rats were used in this study; 12 of which were SHR (ADHD model) from Charles River Laboratories (Wilmington, MA, USA), and 17 were WKY, the normotensive control strain, from Harlan Laboratories (Indianapolis, IN, USA). Rats weighed approximately 175–250 g at the time of testing. Rats were housed in pairs in 47.6 × 20.3 × 26 cm (w × h × d) polycarbonate containers with Alpha Chip bedding material (Northeastern Products Corp., Warrensburg, NY, USA) and had free access to water. One week after arrival, all rats were placed on a restricted food diet of approximately 20 g of standard rat pellets per day (Purina, St. Louis, MO, USA). Rats were fed after their daily behavioral training session. Food restriction continued for the duration of the experiment. All procedures were approved by the Rutgers University Institutional Animal Care and Use Committee.

Behavioral training and testing took place in 12 identical rat operant conditioning chambers (Med Associates, St. Albans, VT, USA). Each operant conditioning chamber measured 30.5 × 24.1 × 21 cm (w × h × d) and was constructed of stainless steel and clear plastic walls and a stainless steel grid floor. A food cup with infrared detectors was centered on one wall of the operant conditioning chamber. Retractable levers were situated to the left and right of the food cup. Responses on these levers delivered one food pellet from a pellet dispenser mounted outside the operant conditioning chamber. Two types of pellets were used in the experimental procedures: 45-mg grain-based pellets and chocolate-flavored purified pellets (Bio-serv, Frenchtown, NJ, USA). Each operant conditioning chamber was housed in a sound attenuating shell and equipped with a ventilation fan that was activated during behavioral procedures. Control over the operant conditioning chambers was enabled by a personal computer operating through an interface. Operant conditioning chamber operation and data collection were carried out with Med Associates proprietary software (Med-PC).

### Behavioral Procedures

#### General Procedures

A timeline of behavioral procedures is depicted in **Table 1**. Behavioral procedures commenced after 1 week of food restriction. Rats were provided with one 15-min session to habituate to the testing chamber, after which they began behavioral training. MPH or saline injections were carried out before devaluation test sessions to examine their effects on choice performance.

**Table 1:** Timeline of behavioral procedures.

	Habituation	Instrumental training	Devaluation test and MPH injection	Instrumental reminder session	Contingency degradation training and test	Locomotor test
Day	1–2	3–12	12–14	15	16–21	22–27


*MPH: Methylphenidate.*

#### Instrumental Conditioning

Rats underwent two training sessions per day; in one session, responses on one lever were associated with delivery of grain pellets and in the other session responses on a different lever were associated with chocolate pellet delivery. For each training session, one lever was inserted into the chamber and responses the rats made on the lever delivered a single food pellet associated with that lever. The session terminated when rats earned 20 pellets or 25 min had elapsed. Rats were trained daily on each lever in separate sessions with a 30-min interval between sessions. Training lasted for 10 days (see **Table 1**); on days 1–3, each response on the lever resulted in pellet delivery (continuous reinforcement). On days 4–5, pellets were delivered according to a variable-ratio (VR) five schedule, which required, on average, five responses to earn a pellet reward. On days 6–8, pellets were delivered according to a VR-15 schedule. On days 9–10, pellets were delivered according to a VR-20 schedule.

#### Outcome Devaluation Test and MPH Injection

Rats were placed in individual cages identical to their home cage and provided with 25 g of chocolate-flavored pellets. After 30 min, rats were given an intraperitoneal injection of MPH hydrochloride (Sigma Aldrich, St. Louis, MO, USA) dissolved in 0.8% saline or, for the control condition, an equal volume of 0.8% saline. MPH dosage was 2 mg/kg body weight diluted to 2 mg/ml. Rats were returned to the cages containing chocolate pellets for an additional 30 min. They were then placed in the operant conditioning chamber and both levers were inserted. Rats had the opportunity to respond on either lever for 10 min. No outcomes were presented in this session. The test was repeated the following day with the same outcome devalued. Rats that received MPH on the first test received saline on the second test, while rats that received saline on the first test received MPH on the second test. Since our results showed that rats had no preference for chocolate or grain pellets (see Results section: “Instrumental Training”), we only devalued one of the two outcomes to control for this variable across medication status (MPH and saline injections). Further, this will prevent overtraining that could result from devaluing each outcome for each medication status (four devaluation sessions for each rat).

#### Contingency Degradation Training

After the devaluation test, rats received two sessions of retraining (one session with each lever) on a VR-20 schedule before the selective degradation of one of the instrumental contingencies (Hammond, 1980). During contingency degradation, responses on each lever continued to deliver the same outcomes as during training. However, one of the two outcomes was also delivered non-contingently; for every second in, which rats made no lever response, there was a 5% probability of dispensing one pellet. For half the animals, the degraded outcome was chocolate pellets, and for the remaining animals it was grain pellets. Non-contingent outcome delivery occurred during all training sessions. Thus, for one lever-training session, the non-contingent outcome was the same as that earned by a response on the lever, whereas for the other lever-training session the non-contingent outcome differed from the contingent outcome. The rats were given two 20-min training sessions each day, one on each lever with a break of approximately one hour between sessions. Training continued for 4–5 days.

#### Contingency Degradation Test

On the day after the final day of contingency training, rats in both groups received a choice extinction test. The test was identical to the choice test following outcome devaluation; it began with the insertion of both levers and the onset of the house light and ended 10 min later with the retraction of the levers and the offset of the house light. No outcomes were presented during this session. We made no injections prior to this test.

#### Locomotor Activity Assay

Rats were individually placed in an activity monitoring arena equipped with an automated locomotor activity detection system (Accuscan, Columbus, OH, USA). Rats were placed in the arena for a 60-min habituation session. Immediately after habituation, rats were injected with saline and returned to the arena for 60 min, followed by a 60-min session with 2 mg/kg MPH injections. A measure of locomotor activity (HACTV: horizontal activity) was collected based on the number of photobeam breaks that occurred as animals moved through the arena.

### Statistics and Data Analysis

For instrumental conditioning tests, the rate of responses was calculated as the number of lever presses per minute during each session. Reinforcer type (chocolate or grain pellet) was collapsed across training sessions, as no effect of reinforcer type was observed on measures of response rate. Responses on the two levers were categorized as devalued or valued for the outcome devaluation test, and degraded and non-degraded for the contingency degradation test. The lever that delivers grain pellets was labeled as valued, and the one that delivers chocolate pellets was labeled as devalued. Similarly, the lever associated with the contingent outcome was labeled as non-degraded, and the lever associated with the non-contingent outcome was labeled as degraded. Data were normalized by dividing responses on the valued or devalued lever by total (valued plus devalued) responses. Normalization was carried out because of strain differences in overall response rates during the tests. MPH and saline injections were intermixed for all experiments; therefore, there was no injection-order effect to influence outcome devaluation responding. For the contingency degradation test, we lost data for one SHR rat due to a technical error. Additionally, food consumption and locomotor activity tests were conducted on 12 WKY rats. Data analysis was conducted using SPSS Statistics Version 20.0. The normality of data distribution was checked using Kolmogorov–Smirnov tests. All data were normally distributed (*p* > 0.1). To analyze instrumental performance we used 2-factor ANOVA and planned comparisons using two-tailed *t*-tests. The level of significance was set at α = 0.05.

## Results

### Instrumental Training

**FIGURE 1:**
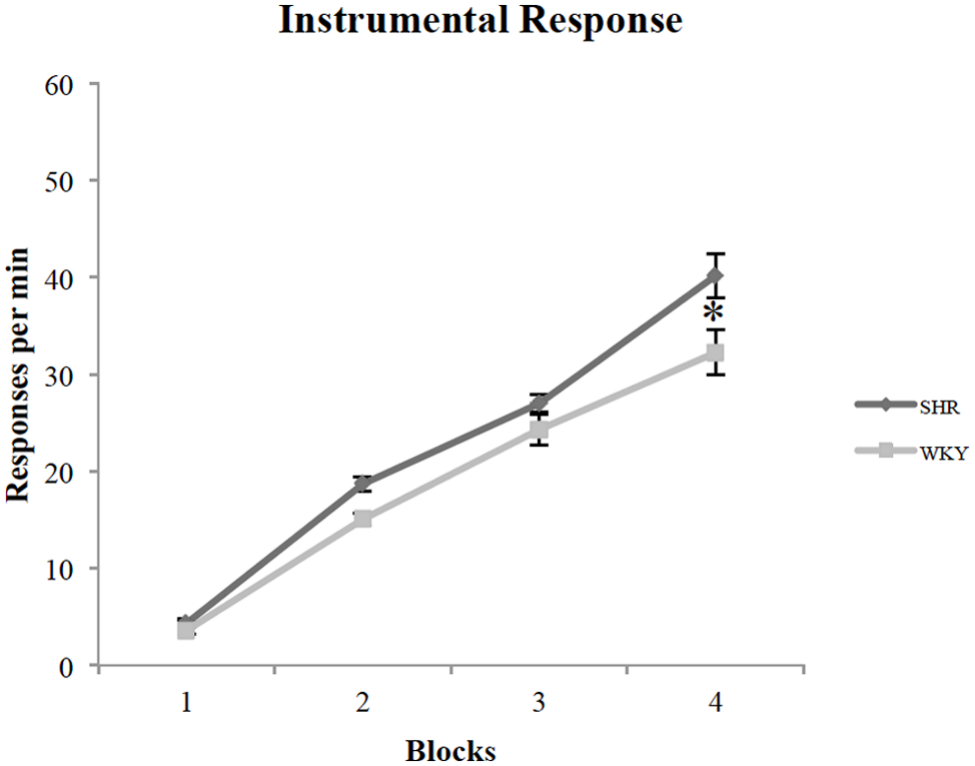
**Performance of SHR and WKY rats on instrumental training blocks (each block represents the average of two training sessions).** The mean number of presses per min on the four blocks of instrumental training in SHR (*N* = 12) and WKY (*N* = 17) rats (error bars = ±SEM) (^∗^*p* < 0.05).

All rats acquired an instrumental response; however, SHR rats exhibited greater response rates across training sessions compared to WKY rats. **Figure 1** represents the lever-pressing rate in SHR and WKY rats. A repeated measures ANOVA confirmed (1) a significant effect of training *Block* [*F*(3,81) = 259.3, *p* < 0.001], (2) a significant effect of *Strain* [*F*(1,27) = 6.7, *p* = 0.015], and (3) a significant *Block ^∗^ Strain* interaction [*F*(3,81) = 3.2, *p* = 0.028]. SHR responses were significantly higher than WKY responses over blocks two and four (*p* = 0.001, *p* = 0.026, respectively, independent-sample *t*-test).

To investigate wither reward type (chocolate vs. grain pellets) had any influence on lever presses, we examined each strain’s responses with the reward type as a factor. A repeated-measures ANOVA revealed that there is no effect of reward type in both SHR [*F*(1,11) = 2.88, *p* = 0.12] and WKY [*F*(1,16) = 0.36, *p* = 0.56] rats.

### Outcome Devaluation Test

**FIGURE 2:**
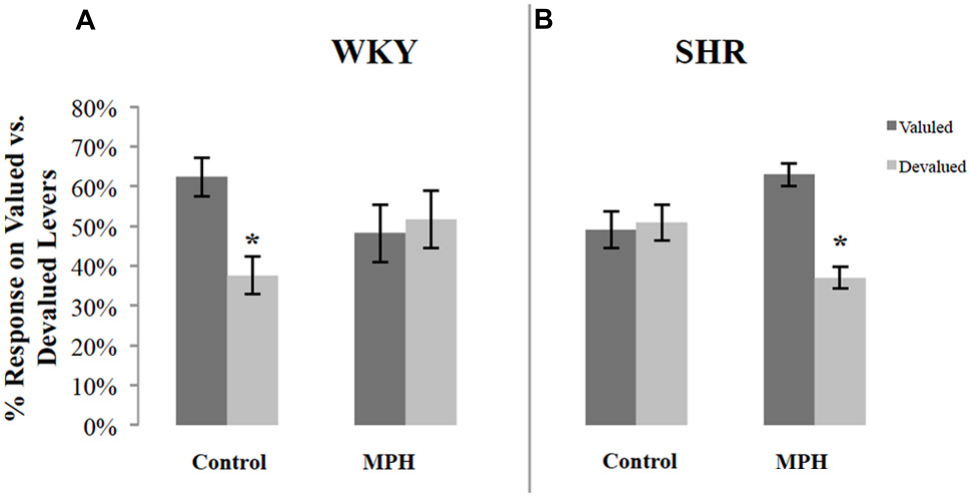
**Normalized performance of SHR and WKY rats during the 10-min devaluation test.** The percentage of responses on the valued and devalued levers for **(A)** WKY (*N* = 17) and **(B)** SHR (*N* = 12) rats (error bars = ±SEM**)** (^∗^*p* < 0.05).

*B*ecause of variability in overall response rates during the choice test, responses on the valuated and devaluated levers were normalized as a percentage of total responses during the test. **Figure 2** illustrates the percentage of responses on the valuated versus the devaluated lever in SHR and WKY rats after saline or MPH injections. Repeated-measures ANOVAs were conducted for each of the two groups tested using outcome value and type of injection as within-subject factors. These analyses revealed significant *Outcome value ^∗^ Injection* interactions among WKY rats [*F*(1,16) = 4.83, *p* = 0.043] (**Figure 2A**) as well as SHR [*F*(1,11) = 12.52, *p* = 0.005] (**Figure 2B**) rats. Following saline injections, WKY rats showed significant goal-directed behavior by responding more on the valuated versus the devaluated lever [**Figure 2A** paired-sample *t*-test: *t*(16) = 2.6, *p* = 0.02]. In contrast, MPH disrupted goal-directed behavior in these rats, as their responses did not differ significantly between valuated and devaluated levers following MPH injection [paired-samples *t*-test: *t*(16) = 0.24, *p* = 0.82].

The reverse pattern was observed in SHR rats. Following saline injections, SHR rats showed no goal-directed behavior, responding equally on the valuated and devaluated levers [paired-sample *t*-test: *t*(11) = 0.2, *p* = 0.84] (**Figure 2B**). MPH restored goal-directed behavior in these rats, as shown by significantly greater responding on the valuated lever compared to the devaluated lever after MPH injection [paired-sample *t*-test: *t*(11) = 4.65, *p* = 0.001].

**FIGURE 3:**
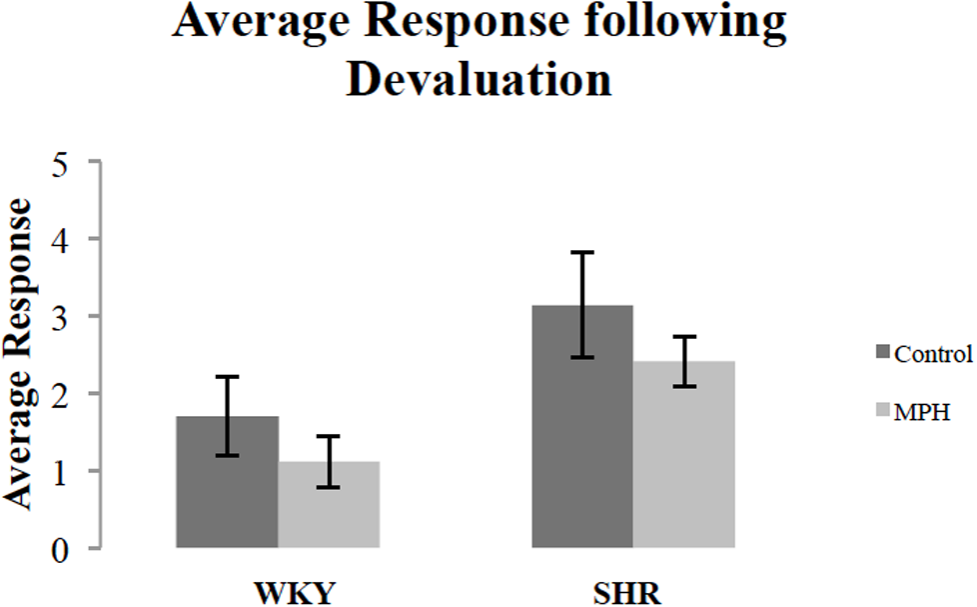
**Overall response rate during the 10-min devaluation test, after receiving methylphenidate (MPH) or saline (Control) injections.** The average of the response rates on both levers for SHR (*N* = 12) and WKY (*N* = 17) rats (error bars = ±SEM) (^∗^*p* < 0.05).

We additionally examined whether MPH injection influenced overall response rates during the devaluation test. **Figure 3** shows the effect of MPH on overall response rates (the average of the response rates on both levers) of both rat strains during the devaluation test after receiving MPH or saline injections. MPH administration suppressed overall response rates. A repeated measures ANOVA showed a significant effect of injection [*F*(1,27) = 4.3, *p* < 0.05], but no strain effect [*F*(1,27) = 3.67, *p* = 0.07], or *Injection ^∗^ Strain* interaction [*F*(1,27) = 0.002, *p* > 0.05]. Although there was a significant main effect of injection, group comparisons were not significant [paired sample *t*-test- WKY: *t*(16) = 1.88, *p* = 0.079; SHR *t*(11) = 1.15, *p* > 0.05, respectively]. Overall, these data indicate that MPH caused rats to modestly suppress instrumental activity during the choice test.

### Contingency Degradation Test

**FIGURE 4:**
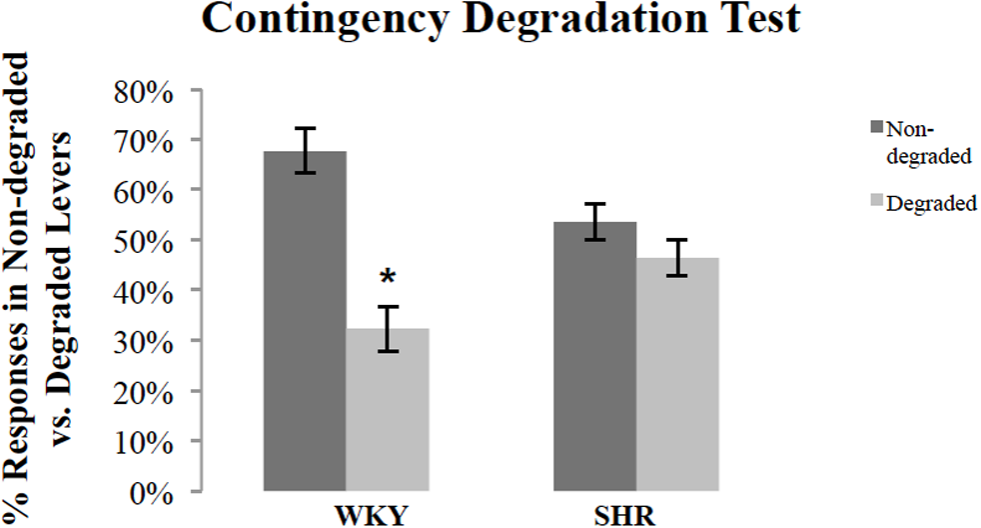
**Normalized response rates during the extinction test after contingency degradation training, shown separately for the action that had the action–outcome contingency degraded and that for which the contingency was not degraded.** The percentage of responses on the degraded and non-degraded levers for WKY (*N* = 17) and SHR (*N* = 11) rats (error bars = ±SEM) (^∗^*p* < 0.05).

Following outcome devaluation, rats underwent contingency degradation training and choice test. **Figure 4** shows response rates, normalized as a percentage of total responses, during the choice test conducted in extinction after contingency degradation training for SHR and WKY rats. A repeated-measures ANOVA revealed a significant main effect of degradation [*F*(1,26) = 11.53, *p* = 0.002] and *Degradation ^∗^ Strain* interaction [*F*(1,26) = 5.12, *p* = 0.03]. A paired-sample *t*-test showed that WKY responses on the non-degraded lever were significantly higher than their responses on the degraded lever [*t*(16) = 3.98, *p* = 0.001]. However, SHR rats did not show any difference between their responses on the non-degraded versus the degraded lever [*t*(10) = 0.98, *p* = 0.35].

### Food Consumption

To determine whether MPH or rat strain influenced food consumption during the devaluation procedure, we examined the amount of food rats consumed during the first 30 min of the devaluation test prior to injections as well as in the 30 min after injections. All rats reached satiety; however, the amount of food required to reach satiety differed by rat strain (**Table 2**). In the 30 min prior to injection, SHR rats consumed a significantly greater amount of food than WKY rats [independent-sample *t*-test: *t*(22) = 3.69, *p* < 0.001].

**Table 2:** Number of food pellets consumed during satiety-induced devaluation.

Injection →	No injection	Normal saline	MPH
Strain ↓	At 30 min	At 60 min	At 60 min
SHR	13.167g ± 0.98	2.667g ± 0.56	1.75g ± 0.75
WKY	9.458g ± 0.21	2.833g ± 0.25	0.75g ± 0.64


*The mean amount of pellets consumed (in grams) (±SEM) in SHR (*N* = 12) and WKY (*N* = 17) rats. MPH, Methylphenidate; SHR, Spontaneously Hypertensive Rats; WKY, Wistar–Kyoto Rats.*

The majority of food consumption occurred in the first 30 min prior to MPH injection (79%). However, MPH altered food consumption in SHR and WKY rats in the remaining 30 min. An ANOVA, using type of injection as a within subject factor, confirmed a significant effect of injection [*F*(1,22) = 25.52, *p* = 0.018]. MPH significantly reduced food consumption in WKY rats [paired-sample *t*-test: *t*(11) = 2.97, *p* = 0.013] but not SHR rats [paired-sample *t*-test, *t*(11) = 0.923, *p* = 0.38]. Overall, these data indicate that SHR rats consumed more food before reaching satiety and that MPH suppressed food consumption selectively in WKY rats.

### Locomotor Activity Test

**FIGURE 5:**
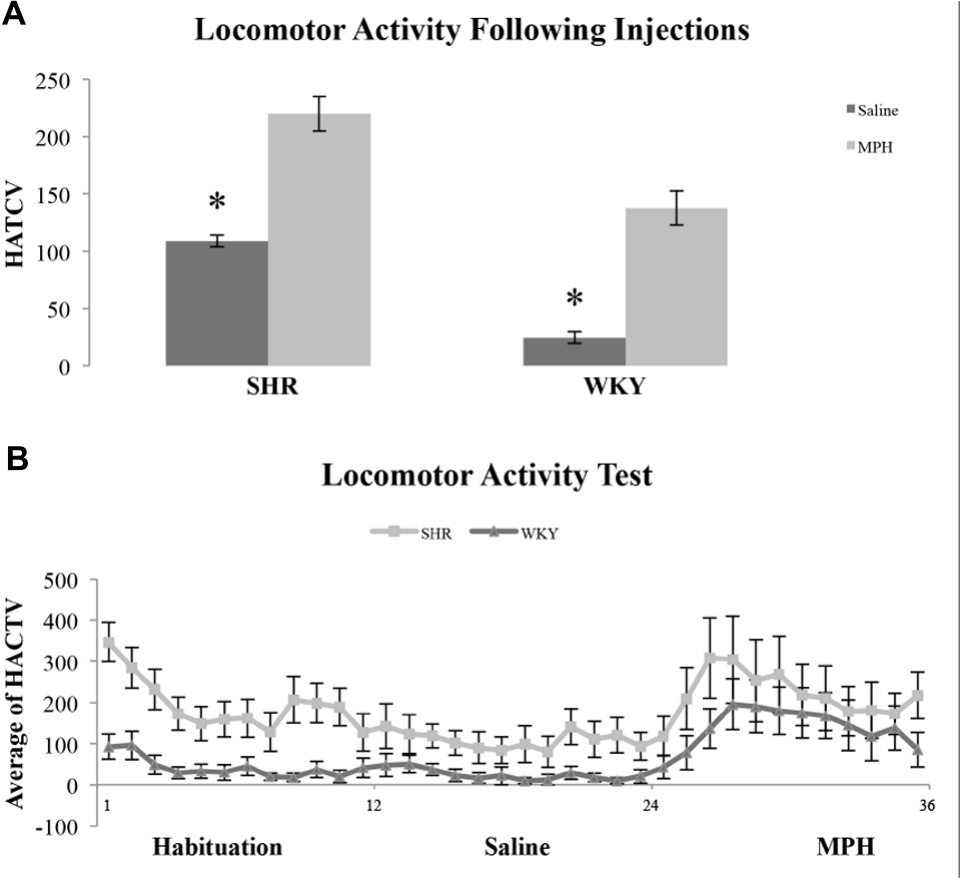
**Locomotor activity in SHR and WKY rats after saline and MPH injections.** **(A)** Locomotor activity expressed as average horizontal activity (HACTV) for one hour after saline and MPH injections SHR (*N* = 12) and WKY (*N* = 12). **(B)** Locomotor activity in SHR and WKY rats in 5-min blocks over (1) habituation phase, block 1–12, (2) saline injection phase, block 12–24, and (3) MPH injection phase, block 24–36 (error bars = ± SEM) (^∗^significant at *p* < 0.05).

We examined locomotor activity to determine whether strain and MPH injection influenced this behavior. We found SHR rats traveled a greater distance as measured by HACTV (see Materials and Methods) and that MPH increased locomotor activity in both strains. HACTV was averaged across 5-min blocks for 1 h after saline and MPH injections. A repeated measures ANOVA on HACTV revealed a significant effect of strain [*F*(1,11) = 26.4, *p* < 0.001] and injection [*F*(2,22) = 13.6, *p* < 0.01]. However, there was no *Phase ^∗^ Strain* interaction [*F*(2,22) = 2.36, *p* > 0.05; **Figure 5B**]. MPH injections did significantly increase HACTV of both strains as compared to saline injection [**Figure 5A**, paired-sample *t*-test- SHR: *t*(22) = 6.45, *p* < 0.001; WKY: *t*(22) = 7.77, *p* < 0.001]. Moreover, HACTV was significantly greater in SHR rats as compared to WKY rats after both saline and MPH injections [**Figure 5B**, independent-sample *t*-test- saline injection: *t*(22) = 11.62, *p* < 0.001; MPH injection: *t*(22) = 3.85, *p* = 0.001].

## Discussion

In this study, we show using outcome devaluation and contingency degradation paradigms that SHR rats have a deficit in goal-directed behavior compared to normotensive WKY rats. Furthermore, we found that deficits in goal-directed action control following outcome devaluation were remediated in SHR rats with MPH administration. In contrast, while goal-directed behavior is well displayed in WKY rats, MPH administration disrupts this behavior. These results suggest that the behavioral phenotype of SHR rats, along with MPH treatment, play complementary roles in determining goal-directed action control.

### Different Patterns of Goal-directed Behavior in WKY vs. SHR Rats and Distinct Effects of MPH

Using an instrumental conditioning paradigm, we present the first experimental evidence of disrupted goal-directed behavior using instrumental procedures in a rat model of ADHD. Both control WKY rats and SHR rats were successful at acquiring an instrumental response, with SHR rats showing a significantly greater response rate during training. Further, using an open field test to evaluate rat locomotor activity, SHR rats showed enhanced locomotor activity as compared to WKY rats. These findings of SHR operant and motor hyperactivity replicate previous research evaluating the use of the SHR strain as a model of ADHD (Wultz et al., 1990; Hill et al., 2012).

Our results suggest a fundamental impairment in goal-directed action control in SHR rats that is remediated by MPH. We used outcome devaluation and contingency degradation paradigms to assess whether animals had formed action–outcome (goal-directed) or stimulus-response (habit) associations. While WKY rats showed goal-directed action control in both paradigms, SHR rats demonstrated a marked deficit in sensitivity to changes in outcome value and to changes in the action–outcome contingency. Lack of sensitivity to these manipulations may reflect either (1) an inability to use action–outcome information to guide choice behavior (i.e., performance deficit) or (2) an inability of SHR rats to learn and/or retrieve action–outcome associations (i.e., learning/memory deficit). We found that treating SHR rats with MPH prior to the choice test following outcome devaluation revealed value-sensitive responding in these animals. SHR rats did encode action–outcome associations during instrumental learning; however, they were only able to use these associations to guide behavior when tested under the effects of MPH. Therefore the deficits we observed in tests of goal-directed behavior in non-medicated SHR rats likely reflects a deficit in performance and not learning of goal-directed actions.

The performance of SHR rats following outcome devaluation is not likely mediated by strain differences in the selective satiety process itself. We did find that SHR rats consumed more pellets compared to WKY animals during the satiety process. Nevertheless, many pellets remained after the 1-h session, by which time all animals had significantly curtailed food consumption, suggesting that all animals had reached a state of satiety. Likewise, although MPH reduced food consumption, it is unlikely that the anorexic effect of MPH altered the devaluation process itself since the majority of food consumption occurred in the 30 min prior to MPH injection. Based on these considerations, we are confident that the selective satiety procedure was equally effective in both SHR and WKY rats, and that the effects of MPH on choice performance were not a consequence of changing the satiety procedure.

To further strengthen our finding of impaired goal-directed behavior in SHR rats, and to exclude any effect of outcome devaluation on this behavior, we used a contingency degradation test after training the rats on a selective degradation of the instrumental contingency. Like the outcome devaluation test, SHR rats were impaired on this test. They performed significantly fewer responses on the lever for which the contingency had been degraded. This result further supports the notion of impaired goal-directed action control in SHR rats.

Overall, our data suggest that SHR rats’ impaired goal-directed behavior is not due to a lack of knowledge of causal consequences or to a failure of the devaluation process prior to the extinction test. This impairment is likely due to a predominance of habitual action control in SHR rats. In contrast, others have reported impaired habit formation and preserved goal-directed behavior in SHR rats (Gauthier et al., 2014). Differences in experimental design and interpretation may explain some of these discrepant results. Finally, one limitation of our study was injecting all rats with the same dose of MPH, while many studies have reported significant variations in the therapeutic doses of MPH. The use of one drug dose limits the conclusions we can draw from this study. A future study exploring the dose-response relationship between MPH and action control is required to fully address this issue.

### Different Neuronal Mechanisms Underlie the Effect of MPH on the Behavior of WKY vs. SHR Rats

Cortico-striatal circuits that include the PFC and striatum mediate goal-directed behavior and habitual learning. Electrophysiological studies using primates (Matsumoto et al., 2003; Matsumoto and Tanaka, 2004) and rats (Mulder et al., 2003) have found neural activity in the PFC related to engagement of specific action–outcome associations. Likewise, lesions of the medial prefrontal cortex result in behavior that is insensitive to changes in outcome value with a stimulus-elicited, rather than goal-anticipated, instrumental responding (Hitchcott et al., 2007). Furthermore, Yin et al. have shown that the dorsomedial striatum plays a critical role in the acquisition and performance of goal-directed actions (Yin et al., 2005), and that the dorsolateral striatum mediates habitual instrumental performance (Yin et al., 2004). Further, they have shown that lesions of the dorsolateral striatum brought normal habitual actions under the control of the goal-directed system. Accordingly, the insensitivity to outcome devaluation in SHR rats might suggest that the neural circuits required for goal-directed actions are dysfunctional in these animals and thus they rely on the habit system instead to control responding.

The effects of MPH on goal-directed behavior likely occur through its modulation of catecholamine availability in the prefrontal cortex and striatum. The therapeutic dose of MPH works primarily via (1) increasing DA signaling through multiple actions, including DAT blockade in the PFC, and significantly enhancing extracellular DA release in the striatum (Volkow et al., 2002; Wilens, 2008) and (2) indirectly increasing NE actions through blockade of its transporters (Berridge et al., 2006). Increasing DA signaling in the PFC enhances goal-directed behavior (Hitchcott et al., 2007). Further, recent physiological studies have shown that NE strengthens network connectivity of the PFC and maintains persistent firing of PFC neurons during working memory tasks through stimulation of postsynaptic α2-adrenoceptors (Arnsten and Dudley, 2005). Thus, the increase in dopaminergic and noradrenergic availability in the PFC of SHR rats might be critical in restoring goal-directed actions by enhancing attentional mechanisms necessary for carrying out goal-directed behavior (Ostlund and Balleine, 2005; Ostlund et al., 2009). However, excessive DA stimulation might cause PFC dysfunction leading to impaired inhibition of undesirable behavior, and a deficit in sustaining attention, as these two behaviors are highly regulated by dopaminergic release in the PFC (Russell, 2003; Brennan and Arnsten, 2008; Arnsten, 2011).

In addition to modulating neurotransmitter release in the PFC, MPH may restore goal-directed behavior through reinforcement-based mechanisms. In the context of DA transfer deficit theory of ADHD, one can argue that administration of MPH restored goal-directed behavior by increasing the magnitude of the anticipatory DA cell firing to predictive cues (pressing the lever) (Tripp and Wickens, 2009, 2012). This is also consistent with the hypothesis that MPH selectively increases the efficacy of conditioned reinforcers (Hill, 1970; Robbins, 1975, 1978). Further studies examining the neural circuits activated in SHR rats during instrumental performance and the site of action of MPH will help to better understand the neural mechanisms underlying altered goal-directed action in ADHD.

## Conclusion

We show for the first time that goal-directed behavior is impaired in a rat model of ADHD and can be remediated in SHR rats with MPH treatment. These results suggest that clinical symptoms exhibited by ADHD patients may reflect impaired goal-directed action control and that MPH may activate this system to re-establish goal-directed behavior.

## Conflict of Interest Statement

The authors declare that the research was conducted in the absence of any commercial or financial relationships that could be construed as a potential conflict of interest.
